# Actin cytoskeleton dynamics in stem cells from autistic individuals

**DOI:** 10.1038/s41598-018-29309-6

**Published:** 2018-07-24

**Authors:** Karina Griesi-Oliveira, Angela May Suzuki, Aline Yasuda Alves, Ana Carolina Cintra Nunes Mafra, Guilherme Lopes Yamamoto, Suzana Ezquina, Yuli Thamires Magalhães, Fabio Luis Forti, Andrea Laurato Sertie, Elaine Cristina Zachi, Estevão Vadasz, Maria Rita Passos-Bueno

**Affiliations:** 1 Hospital Israelita Albert Einstein, São Paulo, Brazil; 2 Departamento de Genética e Biologia Evolutiva, Instituto de Biociência, Universidade de São Paulo, São Paulo, Brazil; 3 Departamento de Bioquímica, Instituto de Química, Universidade de São Paulo, São Paulo, Brazil; 4 Núcleo de Neurociências e Comportamento, Departamento de Psicologia Experimental, Instituto de Psicologia, Universidade de São Paulo, São Paulo, Brazil; 5 Instituto de Psiquiatria do Hospital das Clínicas, Faculdade de Medicina, Universidade de São Paulo, São Paulo, Brazil

## Abstract

Several lines of indirect evidence, such as mutations or dysregulated expression of genes related to cytoskeleton, have suggested that cytoskeletal dynamics, a process essential for axons and dendrites development, is compromised in autism spectrum disorders (ASD). However, no study has yet examined whether cytoskeleton dynamics is functionally altered in cells from ASD patients. Here we investigated the regulation of actin cytoskeleton dynamics in stem cells from human exfoliated deciduous teeth (SHEDs) of 13 ASD patients and 8 control individuals by inducing actin filament depolymerization and then measuing their reconstruction upon activation of the RhoGTPases Rac, Cdc42 or RhoA. We observed that stem cells from seven ASD individuals (53%) presented altered dymanics of filament reconstruction, including a patient recently studied by our group whose iPSC-derived neuronal cells show shorten and less arborized neurites. We also report potentially pathogenic genetic variants that might be related to the alterations in actin repolymerization dynamics observed in some patient-derived cells. Our results suggest that, at least for a subgroup of ASD patients, the dynamics of actin polymerization is impaired, which might be ultimately leading to neuronal abnormalities.

## Introduction

Autism spectrum disorders (ASD) are a group of early onset neurodevelopmental conditions characterized by deficits in social communication skills and restricted or stereotyped patterns of behaviors. According to recent studies, ASD affect 1 in every 68 children and are genetically heterogeneous^1^. In spite of the great advances in molecular tools, that have allowed the identification of the potential causative variants in nearly 25% of the cases, the etiology of ASD remains unknown for the majority of patients^2^.

Genetic studies have revealed that a large number of mutated genes implicated in ASD converge on common biological mechanisms, suggesting that these genetic alterations can lead to similar functional effects and common neurological outcomes^3–5^. One example of such common mechanism is cytoskeleton regulation: recent large-scale CNV and gene expression studies in ASD patients have identified functional groups related to the regulation of actin filaments dynamics, including a global gene expression study conducted by our group^6–10^. Actin filaments are one of the main components of the cytoskeleton and the regulation of their polymerization and depolymerization is essential to neurite outgrowth, dendritic spine formation/plasticity and axonal guidance, which, in turns, sculpt neuronal connectivity^11–13^. Indeed, studies of neurons derived from ASD patients (either from post-mortem brain tissues or from induced pluripotent stem cells), as well as studies using ASD animal models revealed abnormalities in dendrites, axons and in the organization of the neural network^14–17^, and the possible role of defective actin filament dynamics in dendritic spine and synapse formation in ASD pathogenesis has been reviewed elsewhere^18,19^.

Despite this mounting evidence, no functional studies on the regulation of the cytoskeleton dynamics in cells from autistic individuals have been conducted so far. Therefore, the present study aimed to explore the proportion of ASD individuals presenting with abnormal functioning of the actin cytoskeleton dynamics. In order to investigate this, we examined actin filaments reconstitution upon RhoGTPases stimulation (which is the main group of molecules that regulate actin polymerization) in stem cells from human exfoliated deciduous teeth (SHEDs) of ASD patients and control individuals. SHEDs seem to be a suitable cellular model for addressing this question since we have previously shown that genes involved in cytoskeleton regulation are abnormally expressed in SHEDs of autistic subjects^7,17^. Importantly, we also have previously shown that iPSC-derived neurons from one of these patients, who show *TRPC6* haploinsufficiency, present abnormal dendritic spine density and neurite length, which might be due to cytoskeletal dysregulation^17^. Therefore, we included this patient in the present study in order to explore whether altered cytoskeleton dynamics might also be observed in SHEDs derived from this patient. Finally, by using whole-exome sequencing, we looked for genomic alterations in those idiopathic ASD individuals who showed abnormalities in actin cytoskeleton regulation that could be related to this phenotype.

## Results

**Figure 1:**
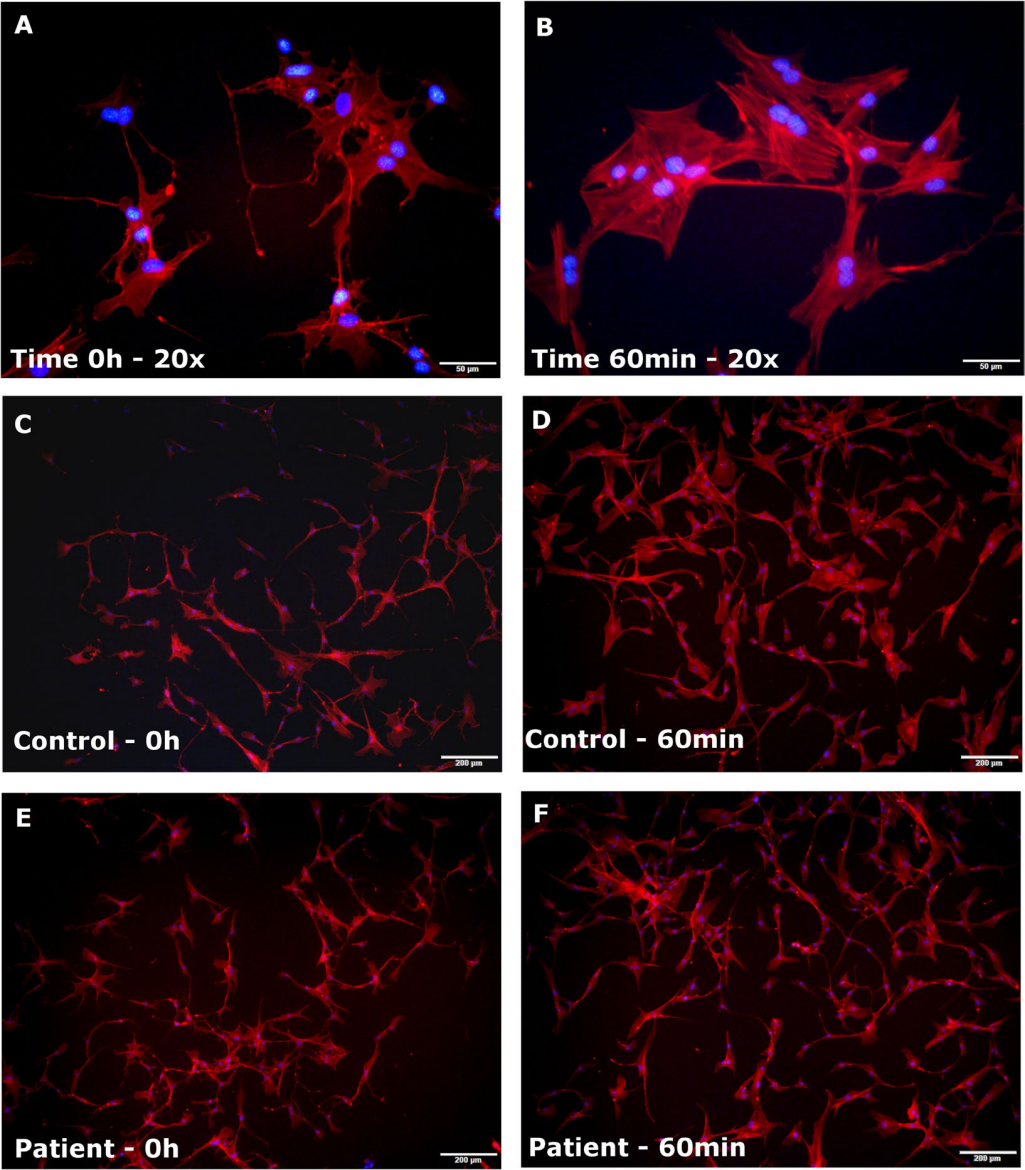
Dynamics of actin re-polymerization on SHEDs. At left, representative images of cells with depolymerized actin filaments right after incubation with ROCKi (time 0 h) at 20x magnification (**A**), for the control sample F5541 (**B**) and for the ASD sample F7511 (**C**). At right, representative images of cells with re-polymerized filaments after 60 minutes of Cdc42 activation treatment at 20x magnification (**D**), for the control sample F5541 (**E**), and for the ASD sample F7511 (**F**). It is possible to notice much more cells with a large cell body due to stress fibers formation in the control sample (**E**), whereas in patient, there are much more cells with a thin cell body with no stress fiber formation (**F**). Scale bar: A and D: 50um; B,C, E and F: 200um.

In order to investigate whether an abnormal dynamics of actin polymerization is observed in ASD, we first promote depolymerization of the actin cytoskeleton in SHEDs derived from an autistic patient with *TRPC6* haploinsufficiency, 12 idiopathic ASD patients and 8 control individuals using Rho kinase inhibitor (ROCKi), which led to depolymerization of filaments in more than 80% of cells from all 21 subjects (Fig. 1A–C; Supplementary Table S1). Next, we promoted actin repolymerization using Direct Activator (DA), a molecule that activates Cdc42, Rac and RhoA simultaneously and directly. DA is a glutamine deaminase molecule that converts glutamine-63 of RhoA and glutamine-61 of both Rac and Cdc42 to glutamate, which blocks intrinsic and GAP-stimulated GTPase activity and results in constitutive activation of these RhoGTPases^20^. We also set up three additional treatment conditions in order to promote activation of RhoGTPases by upstream signals: 100 ng/mL of EGF which promotes the activation of Cdc42^21^, 25 ng/mL of EGF which activates Rac^22^, and 30ug/mL of calpeptin which activates RhoA^23^ (Supplementary Fig. S1A–C). Although activation of Rac and Cdc42 is known to be involved in the formation of lamellipodia and filopodia respectively, we observed the formation of stress fibers in the cell samples treated with EGF, which is indicative of RhoA activation (Fig. S1 C–F). Concordantly, we observed that treatments with both 25 and 100 ng/mL of EGF lead to a later activation of RhoA (Supplementary Fig. S1C). Because stress fibers were abundant and easier to identify than lamellipodia and filopodia, we counted the number of cells that display stress fibers to quantify the percentage of cells with reconstituted actin filaments in all treatment conditions (Supplementary Table 1).

An unsupervised hierarchical clustering analysis divided the samples into two groups according to their behavior over time: in all treatment conditions, control individuals were clustered together within the same group (group 1), whereas ASD patients were distributed across group 1 and group 2, showing that cells from only a subgroup of the patients behaved differentially than those derived from controls (Table 1). Importantly, the cells from ASD individuals included within group 2 respond differently to each type of pharmacological treatment used to induce repolymerization (for example: RhoA activation treatment brought together into group 2 only F2688, F2749 and F3103 samples, while DA treatment clustered F2688, F2749, F2735, F3078 and F3103 samples into group 2).

**Table 1:** Clustering analysis of the samples according to the pattern of actin re-polymerization dynamics.

Group defined by clustering analysis	cdc42	Rac2	RhoA	DA
1	F5541 (C)	F5541 (C)	F5541 (C)	F5541 (C)
	F5594 (C)	F5594 (C)	F5594 (C)	F5594 (C)
	F5618 (C)	F5618 (C)	F5618 (C)	F5618 (C)
	F6119 (C)	F6119 (C)	F6119 (C)	F6119 (C)
	F7647 (C)	F7647 (C)	F7647 (C)	F7647 (C)
	F7672 (C)	F7672 (C)	F7672 (C)	F7672 (C)
	F8370 (C)	F8370 (C)	F8370 (C)	F8370 (C)
	F8564 (C)	F8564 (C)	F8564 (C)	F8564 (C)
	F1740 (P)	F1740 (P)	F1740 (P)	F1740 (P)
	F1850 (P)	F1850 (P)	F1850 (P)	F1850 (P)
	F2613 (P)	F2613 (P)	F2613 (P)	F2613 (P)
	F2709 (P)	F2709 (P)	F2709 (P)	F2709 (P)
	F4289 (P)	F4289 (P)	F4289 (P)	F4289 (P)
	F6281 (P)	F6281 (P)	F6281 (P)	F6281 (P)
	F3103 (P)		F2735 (P)	F6136 (P)
	F6136 (P)		F3078 (P)	F7511 (P)
			F6136 (P)	
			F7511 (P)	
2	F2688 (P)	F2688 (P)	F2688 (P)	F2688 (P)
	F2749 (P)	F2749 (P)	F2749 (P)	F2749 (P)
	F2735 (P)	F2735 (P)	F3103 (P)	F2735 (P)
	F3078 (P)	F3078 (P)		F3078 (P)
	F7511 (P)	F7511 (P)		F3103 (P)
		F3103 (P)		
		F6136 (P)		

P = patient; C = control.

**Figure 2:**
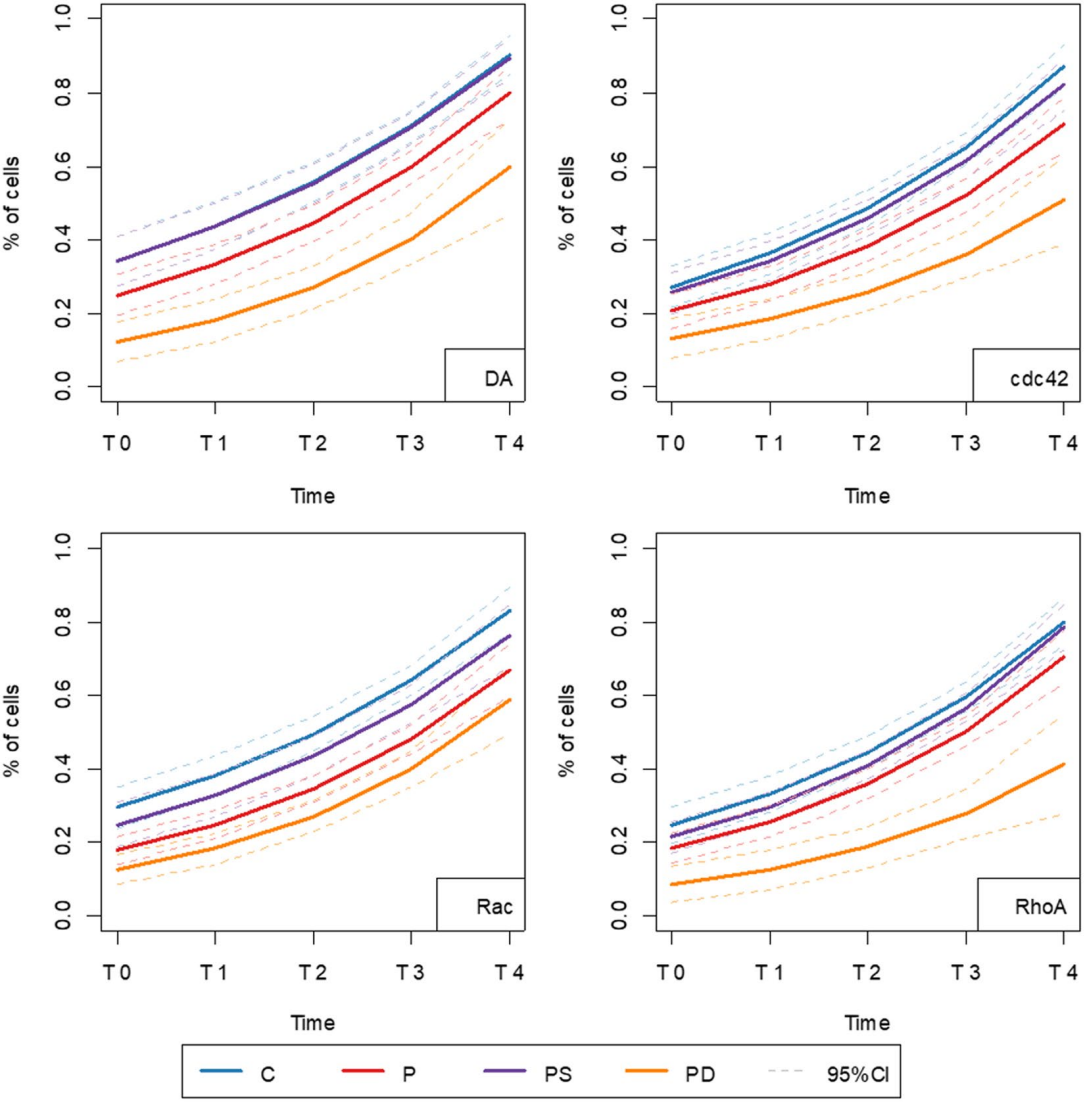
Adjusted models for actin re-polymerization dynamics. Graphs show estimated average of the percentage of cells with reconstituted actin filaments along the time and confidence interval for control (C) samples (blue), all ASD samples (red), ASD samples allocated in group 1 by clustering analysis (purple) or in group 2 (orange) for each treatment. In upper left, it is indicated the p-values obtained by Generalized Additive Model for Location Scale and Shape analysis for each comparison.

To further explore actin repolymerization process over time, we next used a generalized additive model to compare the groups. ASD patients allocated to group 2 showed a significantly lower percentage of cells with reconstituted actin filaments compared to controls (ASD2 X C) in all treatment conditions throughout the observation period (Fig. 2; p-values: DA <0.001; cdc42 = 0.002; Rac <0.001; RhoA = 0.001), while no significant differences were observed comparing ASD patients allocated to group 1 and controls (ASD1 X C) (Fig. 2; p-values: DA = 0.99; cdc42 = 0.686; Rac = 0.236; RhoA = 0.302). On the other hand, when all ASD individuals were compared to controls (ASD X C), patients presented with significantly lower percentage of cells displaying reconstituted networks of actin filaments in DA and Rac activation treatments, although less pronounced than in the comparison ASD2 X C, but did not show differences in both Cdc42 and RhoA activation treatments (Fig. 2; p-values: DA = 0.035, cdc42 = 0.068; Rac <0.001; RhoA = 0.05). This last analysis shows the importance of prior clustering of the samples.

The percentage of cells with filaments still polymerized at the beginning of the experiment (time 0 h) was similar between controls (median: 0.106; interquartile range: 0.06–0.2) and ASD samples that were allocated to group 2 in all treatment conditions (median: 0.115; interquartile range: 0.06–0.14). Thus, we considered that the differences observed between the groups in the course of the repolymerization process were not due to different percentages of cells with intact filaments at time 0 h.

To attest the reproducibility of the results, 64 out of the 336 experimental conditions conducted (21 individuals X 4 repolymerization treatments X 4 time points) were done in duplicate and showed similar results (Intraclass Correlation Coefficient - ICC3 = 0.67; p < 0.0001). Also, 40 experimental conditions were independently evaluated (counted) twice, and the results showed high correlation (ICC3 = 0.85; p < 0.0001).

**Figure 3:**
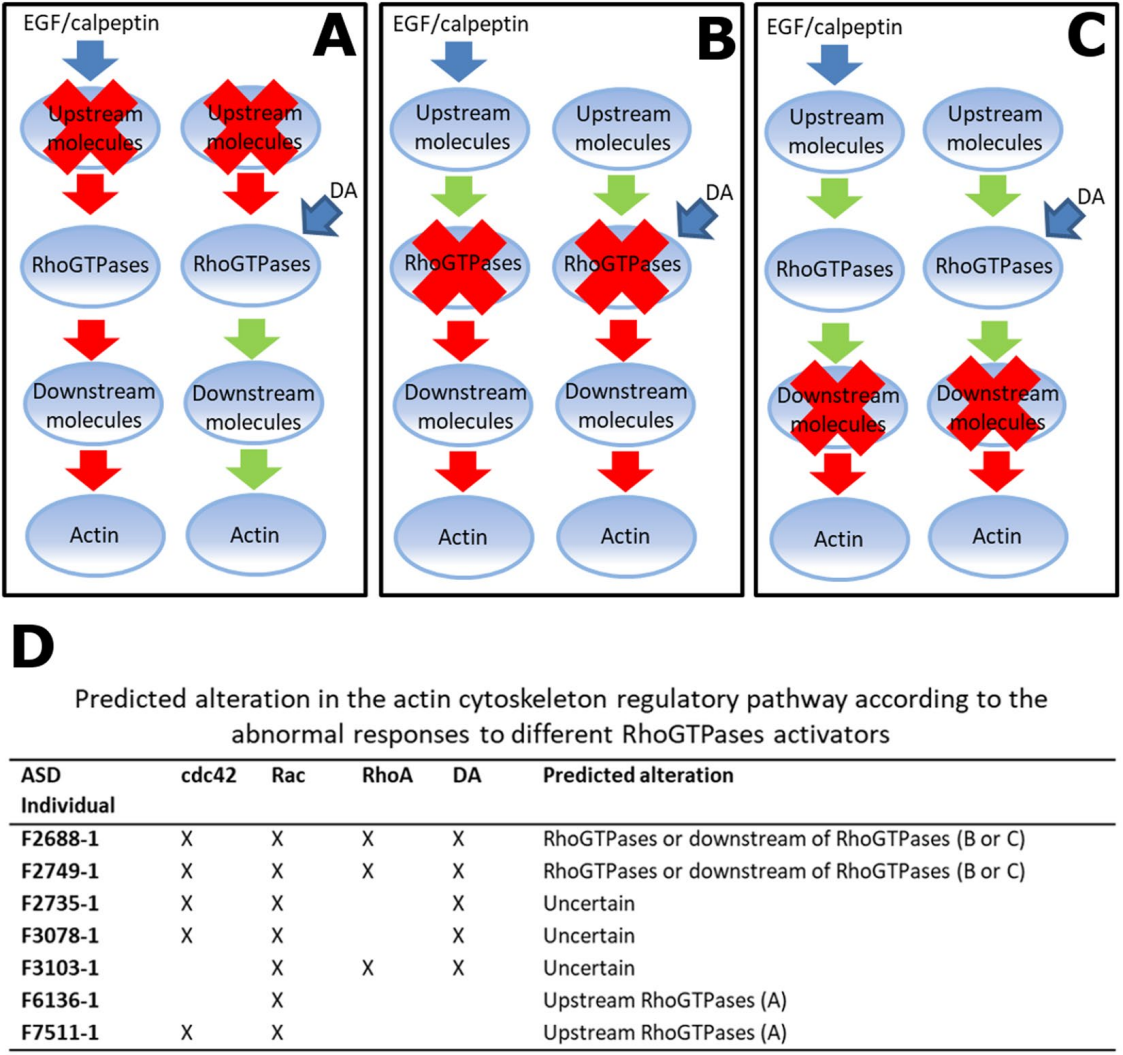
Predicted molecular alterations in actin polymerization regulatory pathway. (**A**–**C**) Schematic representation of possible responses upon treatment with the different activators, depending on the location of a molecular alteration. Red crosses symbolize alteration in any molecule represented inside the balloon. Red arrows indicate abnormal regulation from that point of the pathway to the next, while green arrows, represent normal regulation. (**D**) Summary of the alterations found for each of the ASD individuals that presented abnormal regulation of actin reconstitution in any of the treatments and the predicted alteration in the regulatory pathway accordingly to the model described above.

**Figure 4:**
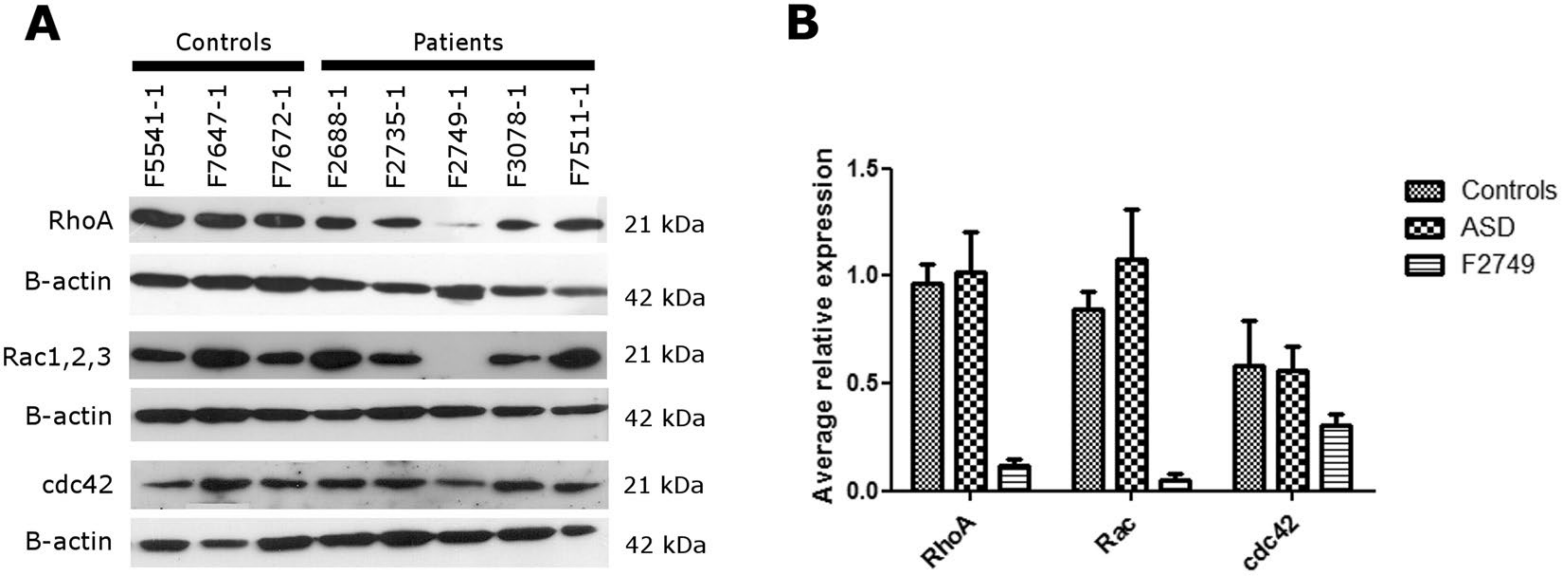
RhoGTPases protein expression. (**A**) Western-blots showing protein expression of RhoA, Rac1/2/3, Cdc42 and b-actin in SHEDs from ASD individuals whose functional assay results indicated an alteration in RhoGTPases or downstream of them (F2688-1 and F2749-1), plus 3 control samples and 3 other ASD samples. (**B**) Average of the relative expression of the RhoGTPases in controls, ASD samples and F2749-1 (RhoGTPases protein levels were normalized by b-actin levels and then normalized to the control sample F7647-1).

By inducing either an upstream activation of RhoGTPases (using EGF and calpeptin) or a direct activation of all of them (using DA) to promote actin repolymerization, we expected to be able to disentangle which is the part of the pathway that might be compromised. That is, if the cells carry genetic alterations that compromise function of upstream components of the RhoGTPases pathway, it would be expected that the treatment with DA would correct the abnormal pattern of actin filaments reconstitution in such cases (Fig. 3A). On the other hand, if genetic alterations are in downstream components of the cascade, or within RhoGTPases, the use of DA would not correct the abnormal pattern of actin reconstitution (Fig. 3B,C). Based on these premises, we could predict which part of the pathway might be altered in four out of seven ASD patients who showed abnormal dynamics of actin filaments reconstitution (group 2): for F6136-1 and F7511-1, the putative alterations were predicted to be upstream of the RhoGTPases; and for F2688-1 and F2749-1, the potential alterations were predicted to be either within or downstream the RhoGTPases (Fig. 3D). Thefore, we next evaluated the protein levels of Cdc42, Rac and RhoA in SHEDs from patients F2688-1 and F2749-1, and we found that the expression levels of the 3 RhoGTPases were lower in cells from patient F2749-1 compared to 3 control individuals and 3 other ASD patients (Fig. 4).

Because F2749-1 patient was genetically characterized previously^17^, we submitted only patients F2688-1, F6136-1 and F7511-1 to whole exome sequencing in order to seek for rare genetic variants in genes related to actin dynamics regulation. All variants considered probably pathogenic in these genes are presented in Table 2. Remarkably, we identified rare and potentially damaging missense variants, according to CADD-score^24^, in genes encoding for either upstream or downstream components of the RhoGTPase cascade which fit in the hypotheses raised by our functional assays: F2688-1 harbors a missense alteration in the *CYFIP1* gene, a molecule that act downstream of RhoGTPases; in F7511-1, we identified a missense variant in *DOCK7*, a guanine exchange factor that activates Cdc42 and Rac^25^; and F6136-1 harbors a missense variant in *ARHGEF18*, a guanine exchange factor that acts on Rac1^26^.

**Table 2:** Single nucleotide variants in genes related to cytoskeleton dynamics regulation in individuals F6136-1, F2688-1 and F7511-1.

Individual	Gene	Exonic function	AA change	CADD Rank	Exac Frequency	Brazilian Frequency
F6136	*LPA*	Nonsense SNV	LPA:NM_005577:exon29:c.G4599A:p.W1533x	35	4 × 10^−5^	N/A
	*KRAS*	Frameshift insertion	KRAS:NM_033360:exon5:c.555dupA:p.C186fs	—	N/A	1 × 10^−3^
	*ARHGEF18*	Missense SNV	ARHGEF18:NM_001130955:exon16:c.C2554A:p.Q852K	31	7 × 10^−4^	8 × 10^−4^
F2688	*NRP2*	Missense SNV	NRP2:NM_003872:exon8:c.G1255A:p.A419T	24.7	3 × 10^−5^	N/A
	*APC2*	Missense SNV	APC2:NM_005883:exon2:c.C17T:p.A6V	32	6 × 10^−4^	N/A
	*CYFIP1*	Missense SNV	CYFIP1:NM_014608:exon29:c.A3368T:p.E1123V	25.2	1 × 10^−5^	1 × 10^−3^
	*EPHA1*	Missense SNV	EPHA1:NM_005232:exon8:c.G1540A:p.V514I	22.7	1 × 10^−4^	N/A
F7511	*ITGB6*	Stopgain SNV	ITGB6:NM_000888:exon14:c.C2245T:p.R749X	40	8 × 10^−6^	N/A
	*TRIO*	Missense SNV	TRIO:NM_007118:exon57:c.C9247T:p.R3083C	24	7 × 10^−4^	1 × 10^−3^
	*PIK3C2B*	Missense SNV	PIK3C2B:NM_002646:exon15:c.G2248A:p.G750S	33	5 × 10^−4^	1 × 10^−3^
	*DOCK7*	Missense SNV	DOCK7:NM_001271999:exon40:c.G5071C:p.E1691Q	23.6	3 × 10^−5^	8 × 10^−4^
	*PIK3R5*	Missense SNV	PIK3R5:NM_001251852:exon9:c.T406C:p.F136L	26	8 × 10^−4^	8×10^−4^

Criteria adopted to select the variants: nonsense, frameshift, splicing or predicted damaging missense variants with a frequency in the population <0.01 (population frequencies were based on ExAC database and on an in-house database composed by 600 Brazilian individuals^38^). Damage prediction was based on CADD-score^24^. All the variants listed are present in heterozygosity in the individuals and were inherited from one of the parents. Parents carrying the same variants are not affected.

## Discussion

Our study using SHEDs of ASD individuals revealed that 53% of them (7/13) showed abnormal dynamics of actin filaments reconstruction in at least one of the tested conditions. For our knowledge, this is the first work that has evaluated the functional aspects of cytoskeleton dynamics in cells of ASD individuals, validating previous evidence from genomic and expression studies, which have already pointed to the relevance of these mechanisms to the pathophysiology of the disease^6–10^. The combined analysis of the responses to direct and upstream stimulation of RhoGTPases allowed us to make some assumptions about where this signaling pathway would be compromised, and we could indicate variants in genes that would fit into these assumptions for some of the individuals. We are aware that the relationship between these mutations and the phenotypic alterations observed must be further validated in SHEDs and, specially, in neuronal cells of the patients, in order to prove their influence on the abnormal regulation of cytoskeleton dynamics. However, some literature evidence that support these relationships are worthy to be discussed.

Individual F2749-1, who has proven neuronal and gene expression abnormalities related to cytoskeleton regulation^17^, showed an abnormal actin reconstitution dynamics in SHEDs in response to all the treatments, indicating an alteration within the RhoGTPases or in downstream components of the pathway. In fact, we found that this individual has a lower level of Cdc42, Rac and RhoA protein expression. This can be related to haploinsufficiency of *TRPC6* in this patient, since this gene codes for a cation channel that regulates activation of the transcription factor CREB^27^ which, in turn, might be regulating RhoGTPases expression.

Our functional data suggested that F2688-1 would have an alteration downstream of the RhoGTPases. We found that his patient harbors a rare missense variant in a downstream component of the pathway, *CYFIP1*, which is potentially pathogenic as predicted by CADD-score^24^. It has been demonstrated that CYFIP1 regulates cytoskeleton remodeling via a Rac1-dependent interaction with the WAVE regulatory complex, modulating dendritic morphology and presynaptic function^28,29^. In a non-simultaneous way, CYFIP1 also interacts with eIF4E and seems to have an impact on the regulation of other mTOR signaling molecules, then regulating translation processes^28,30^. Interestingly, besides the alteration in cytoskeleton dynamics seen in this work, the individual F2688-1 also showed an abnormal response to mTOR activation in a previous study of our group (individual identified as ASD 1 in Suzuki *et al*., 2015)^31^. Moreover, the individual F3078-1, who has a duplication of 15q11-q13 involving *CYFIP1*, also showed an impaired dynamics of actin reconstitution in some of the tested conditions here, and a defective pattern of mTOR activation (individual identified as ASD3 in Suzuki *et al*., 2015). Taken together, these results are consistent with a role of *CYFIP1* alterations in the abnormal phenotypes observed in the present and in the previous functional studies.

Finally, the patients F7511-1 and F6136-1, whose patterns of response to the different treatments indicate the existence of alteration in molecules that act upstream of the RhoGTPases, harbor rare and potentially damaging variants in genes that code for guanine exchange factors, molecules that activate RhoGTPases. F7511-1 has a variant on a domain of DOCK7 that is responsible for activation of Rac1 and Cdc42 (https://www.ncbi.nlm.nih.gov/Structure/cdd/wrpsb.cgi?seqinput=NP_001258928.1), which is in agreement to our results that showed abnormal actin reconstitution in this patient only upon Cdc42 or Rac stimulation. On the other hand, *ARHGEF18*, the gene mutated in F6136-1, can activate Rac1 and RhoA^26^, but this individual only showed abnormal response to Rac stimulation.

Some aspects of the approaches used in this study are worthy of further discussion. First, the use of an unsupervised clustering analysis to group the samples as the initial step of the analysis has captured exactly which individuals had an abnormal behavior, which allowed us to identify statistical differences (or more pronounced statistical differences) between the groups, than in a situation in which the whole ASD group is compared to the controls. Taking into account that ASD is a complex and heterogeneous disorder, this strategy should always be considered in functional assays, since on the contrary, an important difference present only in a subgroup of patients might be missed, as illustrated by our results. Second, exome sequencing in ASD usually leads to the identification of dozens of potentially pathogenic genetic variants (based on population frequency and *in silico* predictions) in every individual, becoming difficult to sort out the most relevant ones. As exemplified by our study, functional assays in accessible cells combined with genomic analysis might contribute for the interpretation and selection of the most likely pathogenic candidate variants, which then can be considered for further investigation in more sophisticated models, such as iPSC-derived neurons. Finally, our results suggest that SHED might be a useful model to capture functional alterations in different pathways already associated to ASD, as cytoskeleton dynamics regulation (as demonstrated in this current study and in Griesi-Oliveira *et al*., 2013) and mTOR signaling (demonstrated in Suzuki *et al*., 2015). As illustrated by the case of the individual F2749-1, functional alterations seen in SHEDs might reflect altered neuronal phenotypes, although we are aware that a larger sample must be analyzed in both models in order to give support to this hypothesis.

Here we presented for the first time functional evidence that a significant proportion of ASD patients has an abnormal cytoskeleton dynamics regulation, an important mechanism involved in dendrite and axon formation, extension and plasticity. We believe that functional approaches as used here can lead to the identification of subgroups of patients that share alterations in a same pathway, which might facilitate the search of drugs to treat ASD.

## Material and Methods

### Patients and Cell lines

Patients were ascertained as previously described^7^. Briefly, patients were diagnosed by physicians at Psychiatry Institute – University of São Paulo, following DSM-IV (Diagnostic and Statistical Manual of Mental Disorders, Fourth Edition) criteria and using an interview based on ADI-R (Autism diagnostic interview revised) or Childhood Autism Rating Scale (CARS). Wechsler Intelligence Scale for Children (WISC) to measure intelligence quotient (IQ) was applied whenever possible. Supplementary Table S2 describes the results of such evaluations, as wells as which experiments each sample was used in. All the ASD individuals included in this study (n = 13) were males and negative for Fragile-X Syndrome. The control sample consisted in 8 male individuals with no history of ASD or any other neurodevelopmental disorder diagnosis. This project has been approved by the Ethics Committee of the Bioscience Institute – University of Sao Paulo (protocol number 1.133.486), where the study was conducted, and was conducted in accordance with all the guidelines and legal regulations. After a complete description of the study, written informed consent was signed by the parents. SHED lineages were obtained as previously described^7,32^ and mycoplasma tests attested that the cells were not contaminated. Briefly, the pulp of the teeth was isolated and digested for 15 minutes in a solution of trypsin at 37 °C. The cells were then cultivated in DMEM/F12 (Thermo Scientific, CA, USA) supplemented with 15% of fetal bovine serum (Hyclone, USA) and 1% of non-essential aminoacids (Thermo Scientific).

### Production of RBD-GST and PBD-GST Fusion Proteins for RhoGTPases Activity Assay

In order to certify that the selected inducers would actually activate RhoGTPases, we conducted an activity assay, to measure GTP-bound Cdc42, Rac and RhoA proteins. The following protocol was adapted from Espinha *et al*., 2016 and Ascer *et al*., 2015^33,34^. E. coli (strain BL21-DE3) bacteria were transformed via thermal shock with plasmids containing the sequence encoding the fusion protein RBD-GST (Rhotekin-Binding Domain-fused to Glutathione S-Transferase) or PBD-GST (PAK1-Binding Domain fused to Glutathione-S Transferase), which was kindly donated by Gary M. Bokoch from Scripps Research Institute, La Jolla, CA, USA. RBD-GST and PBD-GST expression was then induced by adding isopropyl β-D thiogalactopyranoside (IPTG, 0.5 mM final concentration), followed by incubation at 37 °C for 3 h. Bacterial suspensions were then centrifuged and the pellets were lysed and sonicated on ice. After bacterial lyses, the suspensions were centrifuged at 14,000 rpm for 30 min at 4 °C, and the soluble fractions containing the fusion proteins were collected. The soluble fractions were incubated in 250 μL of glutathione-Sepharose 4B resin (GE Healthcare, Pittsburgh, PA, USA). The resin containing the bound fusion protein (beads) was then washed 6 times with wash buffer (50 mM Tris, pH 7.5; 0.5% Triton X-100; 150 mM NaCl; 5 mM MgCl2; 1 mM DTT; 1 μg/mL aprotinin; 1 μg/mL leupeptin; and 0.1 mM PMSF) and centrifuged at 3,000 rpm for 3 min. The beads were resuspended in 5 mL of wash buffer containing 10% glycerol, followed by aliquoting and storage at −80 °C. Quantification was performed on a 13% SDS-PAGE using a BSA standard curve.

### RhoA, Rac1 and Cdc42 GTPases Activity Assay

The following protocol was also adapted from Espinha *et al*.^33^ and Ascer *et al*.^34^. SHEDs protein lysates from a control sample were obtained from 10-mm dishes at approximately 60% confluence that were serum starved for 18 h and then treated 100 ng/ml of epidermal growth factor – EGF (Peprotech, NJ, USA) to activate Cdc42^21^ (http://www.cytoskeleton.com/cn02), 25 ng/ml of EGF to activate Rac1/2/3^22^ (http://www.cytoskeleton.com/cn02) and 30 ug/ml of calpeptin (Tocris, UK) to activate RhoA^23^ (http://www.cytoskeleton.com/cn01). The cells were washed twice with ice-cold PBS and disrupted with RIPA lysis buffer (50 mM Tris, pH 7.2, 1% Triton X-100, 0.5% sodium deoxycholate, 0.1% SDS, 500 mM NaCl, 10 mM MgCl2, 1 mM Na3VO4, 1 mM NaF, 2 μg/ml each of pepstatin, aprotinin and leupeptin, and 1 mM PMSF). Protein quantification was performed using the Bradford (Bio-Rad, Hercules, CA) colorimetric method. Rac1 and Cdc42 activity assay was performed with 250 μg of the total lysate and 25 μg of PBD-GST beads. For RhoA activity assay, 400 μg of the total lysate and 25 μg of RBD-GST beads were used. The lysates were incubated with beads at 4 °C for 90 min under constant rotational mix. The samples were centrifuged at 3,000 rpm for 3 min at 4 °C and washed three times with buffer B (50 mmol/L Tris-HCl, pH 7.2, 1% Triton X-100, 150 mmol/L NaCl, 10 mmol/L MgCl2, 10 μg/mL leupeptin and aprotinin, and 0.1 mmol/L PMSF) and collected via centrifugation at 3,000 rpm for 3 min at 4 °C. RhoA-GTP bound to RBD-GST-Sepharose beads or Rac1-GTP and Cdc42-GTP bound to PBD-GST-Sepharose beads were resolved on a 13% SDS-PAGE, which contained lanes loaded with 25 μg of total lysates as control. The gels were transferred to nitrocellulose membranes (Fig. S2), blocked with 5% nonfat milk in TBS-T (20 mM Tris pH 7.6; 137 mM NaCl; 0.1% Tween) for 30 min at room temperature and then incubated for 4 h at room temperature either using a monoclonal anti-RhoA antibody (1:500, Santa Cruz Biotechnology) or a monoclonal anti-Rac1 antibody (1:500, Santa Cruz Biotechnology), or incubated overnight at 4 °C using a polyclonal anti-Cdc42 antibody (1:500, Santa Cruz Biotechnology). Finally, membranes were incubated with the fluorescent secondary antibodies IRDye 680CW or IRDye 800CW for 1 h and visualized using the Odyssey Infrared Image System (CLx model, LI-COR).

### Functional analysis of actin cytoskeleton dynamics

Experimental conditions used here were based on the protocol described by Puschmann and Turnley, 2010)^35^. Cells were grown in DMEM/F12 (Life Technologies, CA, USA), supplemented with 15% of fetal bovine serum (Hyclone, USA) and 1% non-essential aminoacids (Life Technologies) until reach the confluence in a T25 flask. The cells were then detached, counted and plated in 8-well permanox chamber slide at a density of 5 × 10^4^ cells/cm^2^. Twenty-four hours after plating, serum was washed out and cells were incubated overnight in DMEM/F12 with 100uM of Rho kinase inhibitor (ROCKi - Y-27632; Sigma-Aldrich, MO, USA) in order to depolymerize the actin cytoskeleton. In the next morning (time 0 h), ROCKi was washed out and the cells were incubated for 15, 30, 45 or 60 minutes in DMEM/F12 containing specific activators for 3 different RhoGTPases, as mentioned above. A fourth experimental condition was also performed, using a molecule that directly activates the 3 above mentioned RhoGTPases (Rho/Rac/Cdc42 Activator I; Cytoskeleton Inc., CO, USA), that was called in the text as Direct Activator (DA).

At time 0 h and at 15, 30, 45 and 60 minutes after the re-polymerization treatments, cells were fixed and submitted to immunostaining for actin (F-actin vizualization kit, Cytoskeleton Inc). Approximately 200 cells were analyzed in an Olympus IX51 microscope under a 10x objective for time 0 h and for each time point (15, 30, 45 and 60 min) for all the four treatments (activation of Rac, Cdc42, RhoA or DA), verifying the percentage of cells presenting stress fibers. Counting was done blinded to the status of the disease.

### Statistical Analysis

The results obtained for each re-polymerization treatment were firstly submitted to a non-supervised clustering analysis using the K-means for longitudinal data method (R-package *kml*)^36^, to identify which samples have similar or different behavior. Next, we evaluated the differences on the percentage of cells with reconstituted filaments between the groups obtained by k-means analysis, or between patients and controls. To account for the dependence between repeated measures of the same individual along the time and the beta zero-inflated distribution of the data, we used the Generalized Additive Model for Location Scale and Shape (gamlss package)^37^, a model that allows to consider these characteristics of the data for the analysis. Results are presented as estimated means with 95% confidence intervals and p-values. In order to attest the reproducibility of the results, 64 experimental conditions (experimental condition: one individual tested in a given time point for one of the re-polymerization treatments) were done in replicate and 40 experimental conditions were counted twice. To evaluate the correlation between the duplicates, intraclass correlation coefficients (ICC3) were calculated using a two-way mixed model with subjects treated as random effects (psych package).

### Expression analyses of RhoGTPases

Protein expression levels of Cdc42, Rac1/2/3 and RhoA of 5 patients and 3 controls were evaluated by western-blot. Original uncropped blots are presented in Figure S3. The antibodies used were: anti-RhoA (1:1000 dilution; Cell Signaling, MA, USA), anti-Rac1/2/3 (1:1000 dilution; Cell Signaling, MA, USA), anti-Cdc42 (1:200 dilution; Cell Signaling, MA, USA) and anti-Bactin (1:2000 dilution; Sigma-Aldrich). Quantification of the signal was measured using ImageJ and quantification of RhoGTPases expression levels were normalized by B-actin expression levels. Experiment was done in duplicate. To ensure that experimental conditions would resemble those used in the cytoskeleton reconstruction experiments, cells were plated at the same density and serum starved for 24 h before protein extraction.

### Genomic analysis

Exome sequencing was performed for 3 out of the 13 patients enrolled in this study (F2688-1, F6136-1, F7511-1), following standard procedures. Briefly, genomic DNA was randomly fragmented, generating 150-200 bp fragments. DNA library was enriched for exome sequencing using the Truseq Enrichment kit (Illumina, CA, USA) and sequenced on Hiseq2500, generating 100 bp paired reads. Mean coverage was 100x. Burrows-Wheeler Aligner (BWA) algorithm was used to align the sequences and SNVs were identified with SOAPsnp. We limited our analysis to genes related to actin cytoskeleton regulation according to canonical pathways on Ingenuity Pathways Analysis database (from nov/2016). The pathways selected were: Actin Cytoskeleton Signaling, Regulation of actin-based motility by Rho, RhoA Signaling, cdc42 Signaling and Rac Signaling. A genetic variant was considered as potentially pathogenic if: (a) it was a nonsense, frameshift, splicing or a missense variant predicted to be damaging; and (b) it has a frequency <0.01 on ExAC database, as well as on a database of 600 Brazilian controls^38^. As a predictor of damaging potential of the missense variants, we used the Combined Annotation Dependent Depletion (CADD), a method that integrates different annotations into a single score^24^. For variant inclusion, we considered only CADD-scores equal or higher than 20 (a score of 20 indicates that the variant is among the top 1% most damaging variants of all possible substitutions of the human genome).

## Electronic supplementary material

Supplementary Figures

Supplementary Tables 1 and 2
