# Supplementary material for: Actin cytoskeleton dynamics in stem cells from autistic individuals
Source: Sci Rep. 2018 Jul 24;8:11138. doi: 10.1038/s41598-018-29309-6 (PMC6057935; doi:10.1038/s41598-018-29309-6)
Supplement: Supplementary file 1 — Supplementary Figures [file 41598_2018_29309_MOESM1_ESM.pdf]

Supplementary Material

Actin cytoskeleton dynamics in stem cells from autistic individuals

Authors: Karina Griesi-Oliveira[*1,2]; Angela May Suzuki[1]; Aline Yasuda Alves[2]; Ana Carolina Cintra Nunes Mafra[2]; Guilherme Lopes Yamamoto[1]; Suzana Ezquina[1]; Yuli Thamires Magalhães[3]; Fabio Luis Forti[3]; Andrea Laurato Sertie[2]; Elaine Zachi[4]; Estevão Vadasz[5]; Maria Rita Passos-Bueno[1].

1. Departamento de Genética e Biologia Evolutiva, Instituto de Biociência, Universidade de São Paulo, São Paulo, Brasil

2. Hospital Israelita Albert Einstein, São Paulo, Brasil

3. Departamento de Bioquímica, Instituto de Química, Universidade de São Paulo, São Paulo, Brasil

4. Núcleo de Neurociências e Comportamento, Departamento de Psicologia Experimental, Instituto de Psicologia, Universidade de São Paulo, São Paulo, Brasil

5. Instituto de Psiquiatria do Hospital das Clínicas, Faculdade de Medicina, Universidade de São Paulo, São Paulo, Brasil

Address for correspondence:

Karina Griesi-Oliveira, karina.griesi@einstein.br; Av. Albert Einstein, 627, São Paulo – SP, Brazil; CEP 05652-900.

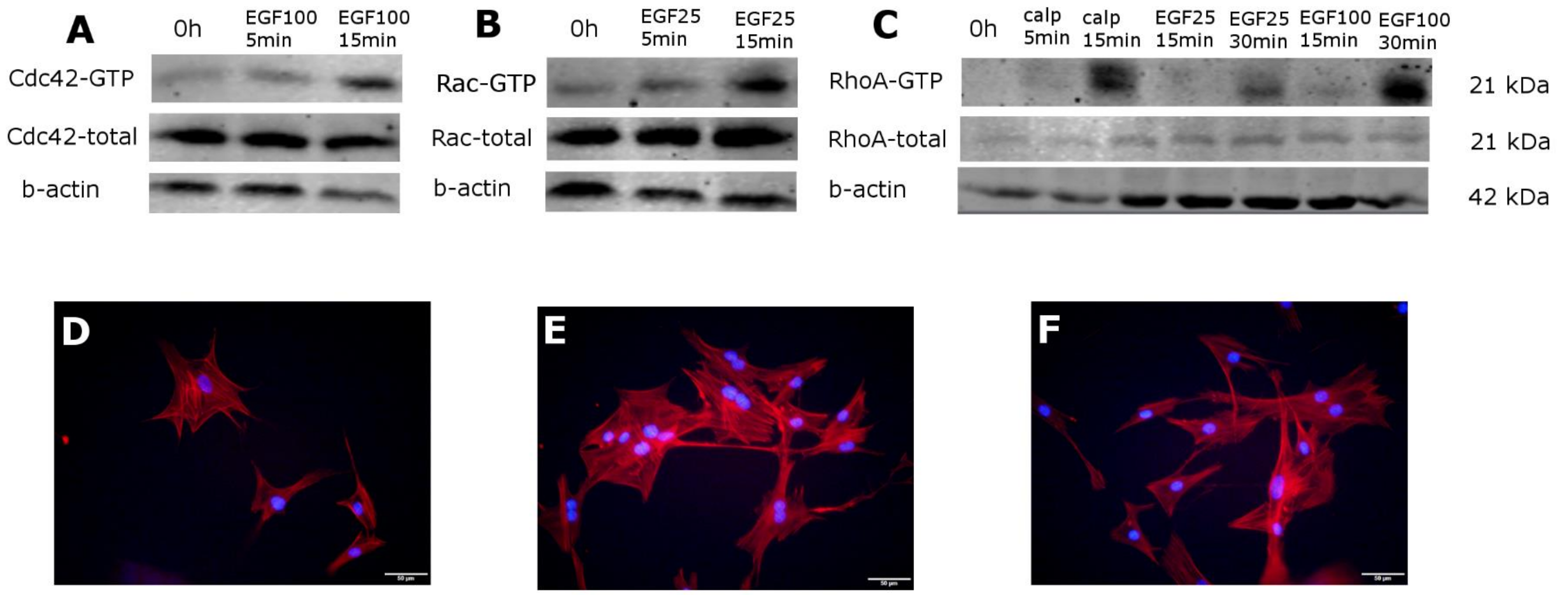

Figure S1. Activation of RhoGTPases and stress fibers formation. Treatment of the cells with 100ng/ml of EGF leads to the activation of cdc42 (A), 25ng/ml of EGF leads to the activation of Rac (B), and 30ug/ml of calpeptin leads to the activation of RhoA (C). However, treatment with EGF for activation of cdc42 or Rac also leads to RhoA activation in a later phase, which is in agreement with the formation of stress fibers (D and E), as also observed in the treatment with calpeptin (F). Scale bar:50um.

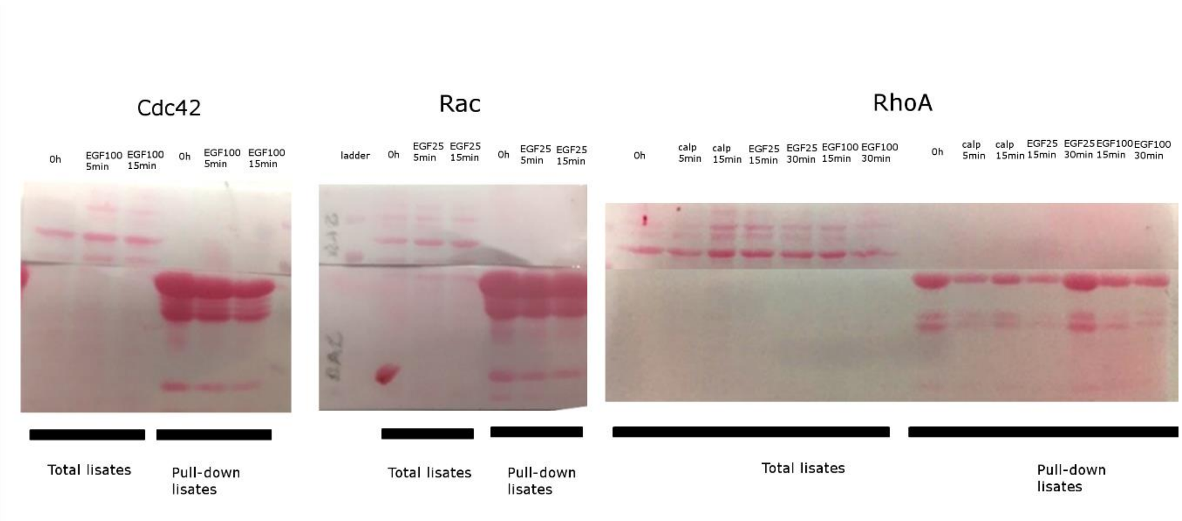

Figure S2. Ponceau staining of nitrocellulose membranes containing total lisates and samples that were submitted to pull-down assay, related to Fig. S1.

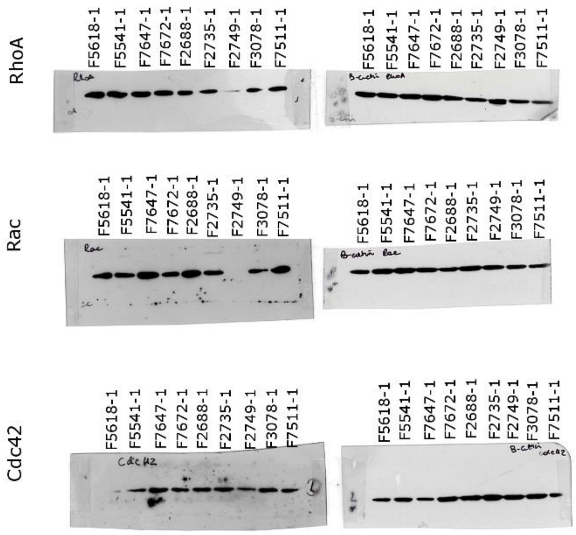

Figure S3. Original uncropped blottings of RhoGTPases quantification assays, related to Fig. 4.
